# Perspectives on Molecular Mechanisms of Hydrate Formation and Growth at Interfaces: A Mini-Review

**DOI:** 10.1021/acs.energyfuels.5c00942

**Published:** 2025-05-08

**Authors:** Anh Phan

**Affiliations:** School of Chemistry and Chemical Engineering, Faculty of Engineering and Physical Sciences, 3660 University of Surrey, Guildford, Surrey GU2 7XH, United Kingdom

## Abstract

Hydrate-based engineering applications hold significant promise due to their physical feasibility and low energy consumption. However, key challenges—including operating conditions, formation and growth rates, and gas storage capacity—continue to impact their viability as sustainable technologies. This mini-review offers key insights into molecular mechanisms governing hydrate nucleation and growth at guest–water interfaces, specifically examining the role of mass transfer thermodynamics across the interface in either promoting or inhibiting gas hydrate formation. Additionally, this review highlights recent advancements, emerging research opportunities, and potential commercialization pathways for these technologies. With continued development, technologies utilizing hydrates have the capability to play a transformative role across multiple industries, offering a more sustainable alternative to existing commercial solutions.

## Graphical abstract

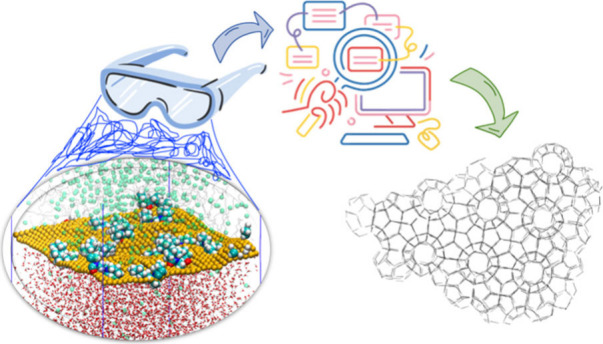

## 1 Introduction

Clathrate hydrates have solid and ice-like structures which occur naturally or could be artificially induced within a specific range of temperature and pressure in a guest–water mixture. In these structures, water molecules create a crystalline lattice through hydrogen bonding, trapping gas molecules inside, and are referred to as clathrate hydrates.^1^ These compounds have become a focal point in sustainable chemistry research due to their potential applications across various scientific and industrial fields, such as the long-term sequestration of carbon dioxide through gas hydrate formation beneath the ocean.^2^ Initially, gas hydrate research principally focused on flow assurance, aiming to prevent blockages in hydrocarbon and gas pipelines caused by hydrate formation.^3–11^ Nevertheless, in recent years, interest in this field has surged, driven by the expanding potential of hydrates in areas such as gas storage, carbon dioxide capture and storage, water desalination, gas separation, transport, and refrigeration, and energy recovery.^2,12–20^

Recent experimental findings, supported by theoretical studies, indicate significant potential not only for improving flow assurance but also for greatly expanding the range of hydrate-based applications toward the water–energy–environment nexus (see Figure 1).^2,18,20,21^ Achieving these advancements requires the development of enabling technologies and the establishment of a comprehensive strategic plan. This progress depends on interdisciplinary collaboration among researchers in various fields. Such collective efforts are essential to unlocking the role of gas hydrates in promoting a cleaner environment while contributing to economic and sustainable development.^2,21^ For example, encapsulating natural gas within gas hydrate structures offers several advantages over conventional compression or liquefaction methods. However, the stringent conditions governing thermodynamics of hydrate formation remain a significant barrier to the large-scale adoption of solidified natural gas technology. To address this, numerous studies^22,23^ have employed thermodynamic modeling software such as Multiflash, CSMGem, and PVTSim to calculate gas hydrate phase equilibria and estimate storage capacities. These simulations typically utilize the van der Waals and Platteeuw model^24^ in combination with various cubic equations of state.^23^ Their results—covering hydrate phase behavior and gas storage capacity—have been validated experimentally^23^ and supported at the molecular level through advanced techniques such as the direct coexistence method, free energy calculations, and hyperparallel tempering.^14,25,26^

**Figure 1:**
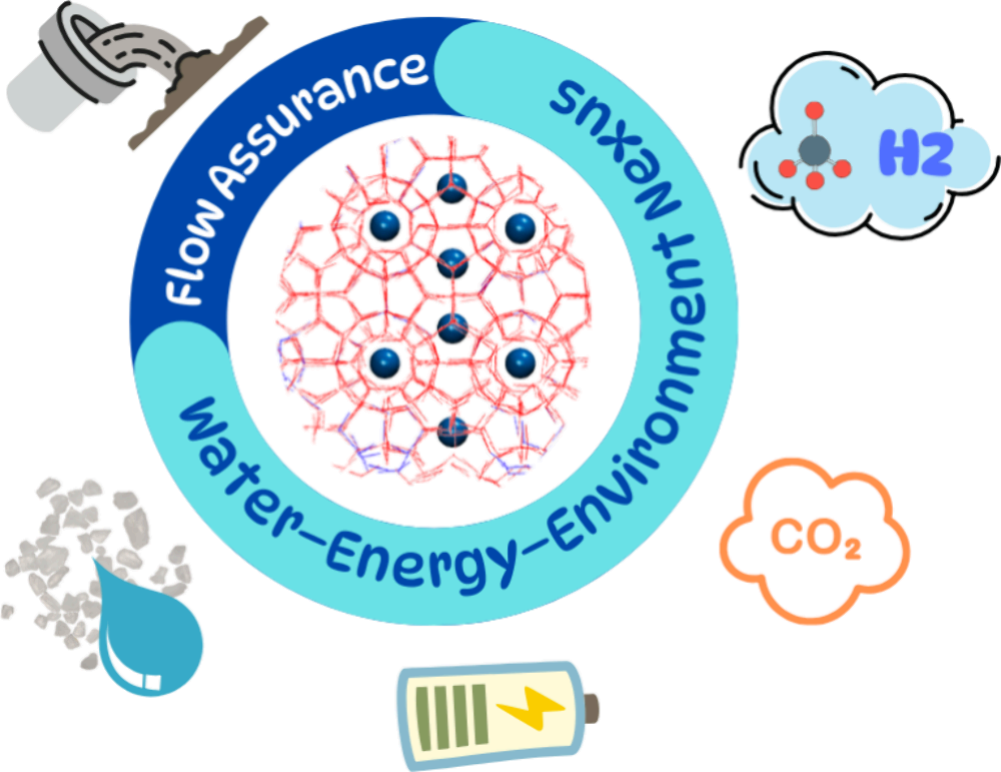
Gas-hydrate-based engineering applications.

Similarly, hydrate-based carbon capture storage (HCCS) has shown promise in both theoretical and experimental studies, demonstrating higher energy efficiency, environmental compatibility, and simplicity compared to conventional CCS technologies.^27^ Despite these benefits, slow kinetics and limited industrial experience remain challenges. The use of surface-active chemicals (SACs),^28–31^ known as hydrate promoters, has proven effective in accelerating hydrate formation kinetics. Conversely, certain SACs can act as inhibitors to enhance flow assurance.^5–8,32–37^ The selection of hydrate promoters/inhibitors typically depends on cost, required dosage, and desirable properties such as noncorrosiveness, nontoxicity, and environmental friendliness. Recent studies have highlighted the effectiveness of green and biobased kinetic promoters, e.g., amino acids,^38,39^ in significantly enhancing hydrate formation and growth. However, the precise mechanisms by which these promoters/inhibitors—including amino acids—affect hydrate processes are still not fully understood. Although hydrate-based technologies offer notable environmental advantages, such as energy-efficient and low-emission gas storage, their role in the context of climate change remains a topic of concern.^40,41^ One widely studied scenario suggests that rising global temperatures could destabilize natural gas hydrate deposits, releasing methane into the atmosphere. This methane release could further amplify greenhouse warming, potentially triggering a feedback loop of continued hydrate dissociation.^2^

Given the complexity and wide range of possibilities in gas hydrate research, a fundamental understanding of their underlying science is crucial for advancing research and enhancing their practical applications for societal benefit. Gaining insights into the microscopic processes of hydrate formation and identifying the fundamental mechanisms regulating their growth can lead the design of targeted engineering solutions that either promote or suppress hydrate formation as needed. Ultimately, the purpose of modeling and experimental studies is to develop improved techniques for managing hydrates—whether as challenges, particularly in the petroleum and gas sector, or as opportunities for new industrialized applications that leverage gas hydrates for engineering advancements.

Multiple theories have been suggested to elucidate the microscopic mechanism of clathrate hydrate nucleation.^42^ Most of the supporting and opposing evidence for these theories comes from computational modeling, because the crystalline nuclei are typically extremely small to be directly observed through experiments.^43^ Nevertheless, a range of diffraction and spectroscopic techniques^44–50^ has been employed to study the nucleation process and provide experimental evidence supporting theoretical models. For example, differential scanning calorimetry was employed to investigate hydrate nucleation in gas-phase hydrate formers.^45^ In situ Raman spectroscopy was applied to examine clathrate morphology, measure guest concentrations in the residual gas phase, and analyze structural changes at the guest–water interface, offering insight into nucleation and crystallization mechanisms.^44,47,48^ Similarly, nuclear magnetic resonance (NMR) spectroscopy was utilized to monitor hydrate nucleation and growth on ice surfaces and within various guest–water mixtures.^49^ More recently, in situ synchrotron X-ray diffraction experiments were conducted to study the formation of methane–carbon dioxide gas hydrates under high vacuum conditions at moderately low temperatures.^50^

One of the earliest hypotheses, known as the labile cluster hypothesis, was introduced by Sloan and colleagues.^51,52^ It suggests that water molecules in solution form cage-like clusters resembling the polyhedral structures of clathrates around guest molecules, which then merge within the liquid phase to create the crystal’s unit cell. However, molecular modellings have since demonstrated that individual clathrate cages, whether empty or guest-filled, are rare in solution and only exist fleetingly for a few picoseconds.^53^ Further studies by Trout and Radhakrishnan^54^ calculated the energy barrier for carbon dioxide-filled cage aggregation and found that these cages were more likely to disintegrate than to merge and form a stable crystal nucleus. Guo and colleagues^55^ analyzed 60 million methane hydration shells through molecular simulations, revealing that fully enclosed polyhedral cages form exclusively in solutions with a high methane concentration, with a likelihood of 10^–6^.^55^ In a separate work, Guo et al.^56^ showed that methane molecules tend to adsorb onto dodecahedral cages and that the stability of these cages enhances as more solute molecules surround them. Based on these findings, it has been speculated that if the labile cluster hypothesis holds any validity, the clusters are likely not isolated cages but rather aggregates involving multiple guest molecules. The latest hypothesis, suggested by Trout and Radhakrishnan,^54^ is the local structuring hypothesis. This theory suggests that the rate-limiting step in hydrate nucleation is a concentration variation that organizes guest molecules into a configuration resembling that of a hydrate crystal. According to this model, water molecules then align in response to the guest molecules, forming the cages that make up the hydrate nucleus. This mechanism remains widely supported by the latest computational studies.^57–60^

With the limited number of hypotheses proposed for hydrate nucleation, it is unlikely that a single universal mechanism applies across different conditions and hydrate-forming environments. Hydrate nucleation—particularly primary heterogeneous nucleation, suggested by the study of Peters et al.^61^ under realistic conditions—is fundamentally an interfacial phenomenon driven by guest–host interactions and free energy fluctuations.^28,57,62^ The guest–host interface, owning to its appropriate thermodynamic conditions, presents the greatest likelihood for initiating the formation of small hydrate embryos.^42^ Gaining a deeper understanding of the dynamic interactions at these interfaces across coexisting phases is essential for comprehending the broader nucleation process. Advancing this understanding will require more sophisticated experimental equipment and imaging technologies capable of providing high-resolution, in situ observations of interfacial properties. When direct experimental investigation is challenging, molecular dynamics (MD) simulations may serve as an alternative, albeit with limitations, to offer insights into hydrate nucleation mechanisms.^42,58,60,62^ Since nucleation marks the very beginning of hydrate formation, any advancements in this field could drive progress in both hydrate research and engineering, allowing for the controlled acceleration or suppression of nucleation depending on the application.

Following nucleation, hydrate growth requires efficient mass transfer of water and guest molecules to the hydrate surface, involving gas transport from the guest–water interface into the aqueous phase, and subsequently to the hydrate interface.^2,63^ Similarly, there is unlikely to be a comprehensive framework for hydrate growth, as various factors—such as fluid composition, water content, and multiphase flow conditions—affect growth through mass and heat transfer, reaction kinetics, or a combination of these processes.^28,62,64,65^ Rather than incorporating an extensive number of variables, the focus should be on evaluating specific systems to determine whether promoting or inhibiting hydrate growth is preferable. Careful examination of mass transfer at guest–water interfaces is key to comprehending hydrate growth kinetics. Computational and experimental studies^62,66–68^ have shown that hydrate films grow in thickness at these interfaces through continued mass transfer, leading to further hydrate formation. Unlike nucleation, hydrate growth occurs at meso- or macroscopic levels, where real-time monitoring of the evolving hydrate phase can provide valuable insights into its kinetics, morphology, and interactions with surrounding phases.^66,69,70^

Recent decades have seen growing emphasis on the guest–water interface in hydrate research, aiming to enable predictive control of nucleation and growth in natural and engineered systems—an essential step toward advancing hydrate-based technologies.^11,71^ In this review, we critically examine simulation studies that investigate the molecular mechanisms of hydrate formation and growth at interfaces, complemented by experimental evidence where available. Particular emphasis is placed on the role of mass transfer thermodynamics in advancing our understanding of these processes. By synthesizing and generalizing findings from the literature, this review aims to contribute to a more comprehensive and universal perspective on hydrate formation at interfaces. The insights presented may support the rational design of SACs that act as hydrate promoters/inhibitors across a wide range of hydrate-related applications. Each incremental advancement in hydrate-based technology and science is significant, collectively bringing the use of gas hydrates—as a reserve, an engineering solution, and a technological platform—closer to reality.

## 2 Hydrate Formation at Guest–Water Interfaces

Structural fluctuations in a metastable guest–water mixture play a crucial role in driving nucleation events.^72^ Therefore, understanding the characteristics of metastable guest solutions is essential for gaining deeper insights into hydrate nucleation and the memory effect. A key aspect of these solutions is guest oversaturation, making the study of solubility of guest molecules an important part of this process.^72^ However, while extensive experimental solubility data exist in thermodynamic equilibrium states, equivalent analysis for metastable states remain scarce.^72^ Specifically, the threshold of methane supersaturation required for hydrate formation remains uncertain.

To investigate the key mechanisms of hydrate formation at guest–water interfaces, Nase et al.^73^ conducted X-ray reflectivity measurements (see Figure 2, top panel). Their findings indicate that guest molecules gather in a highly local area just beneath the interface, forming a distinct layer that signifies supersaturation relative to the guest–water phase. In Figure 2, bottom panel, Nase et al.^73^ illustrated this phenomenon through an electron density distribution of the layer during formation, alongside a diagram illustrating the buildup of guest molecules beneath the interface. In contrast, no layer formation or hydrate formation was observed in experiments examining the liquid alkane–water interface. However, experiments with gaseous xenon consistently resulted in hydrate formation, accompanied by the existence of a supersaturated interfacial layer in every trial. Similarly, for liquid carbon dioxide, a layer was observed to form immediately before hydrate formation occurred. Based on these observations, Nase et al.^73^ concluded that a supersaturated layer of guest molecules is a prerequisite for hydrate formation in stable two-phase systems. This supersaturation significantly elevates the local density of guest molecules, thereby enhancing the likelihood of hydrate formation. Subsequent MD simulations by English et al.^74^ supported these findings, indicating that slow methane diffusion from the interface leads to increased aggregate density within the aqueous phase and promotes methane cluster formation.

**Figure 2:**
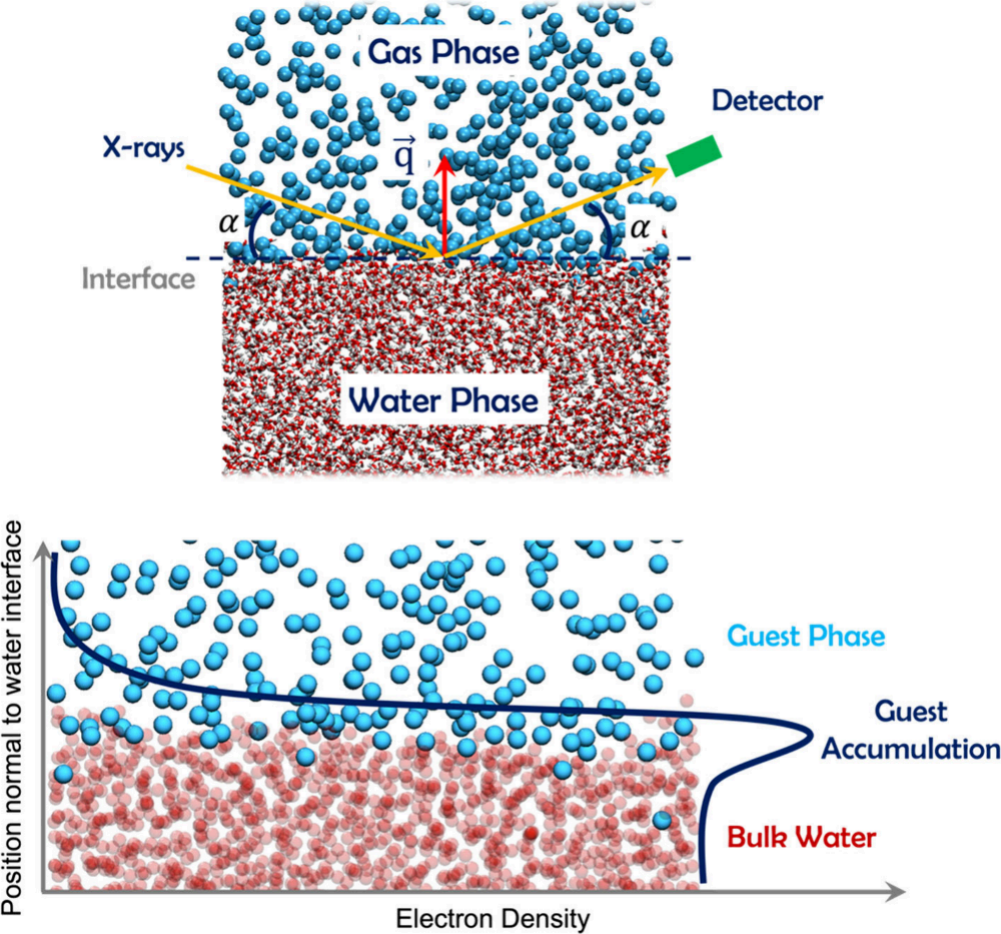
*Top*: Molecular-level depiction of a representative interface under study, along with a schematic illustration of the X-ray scattering setup. *Bottom*: Electron density profile and conceptual illustration of the guest-concentrated interfacial layer. Reproduced from Nase et al.^73^ Copyright 2012 American Chemical Society.

## 3 Morphology of the Guest–Water Interface on Gas Solubility

Walsh et al.^75^ examined the induction of methane hydrate nucleation under different thermodynamic conditions and interfacial curvatures (see Figure 3, top). As anticipated, methane solubility increases with higher pressure and lower temperature. Furthermore, solubility is greater with increased interfacial curvature, following the order: flat < cylindrical < spherical (see Figure 3, bottom left). This increase in solubility is attributed to the increased effective pressure in the methane phase, as explained by the Laplace–Young equation:^76^ *ΔP* = 2γ/*R* (1), where *ΔP* = *P*_vap_ – *P*_liq_ represents the pressure gradient across the bubble interface between the vapor phase and surrounding medium, *R* is the bubble radius, and γ is the surface tension. According to the Laplace–Young equation, as *R* decreases–indicating enhanced interfacial curvature (*R* → ∝ corresponds to the flat surface)–the pressure of the gas above the liquid increases.^77^ Therefore, the increase in methane solubility is directly linked to the increase in interfacial curvature.

**Figure 3:**
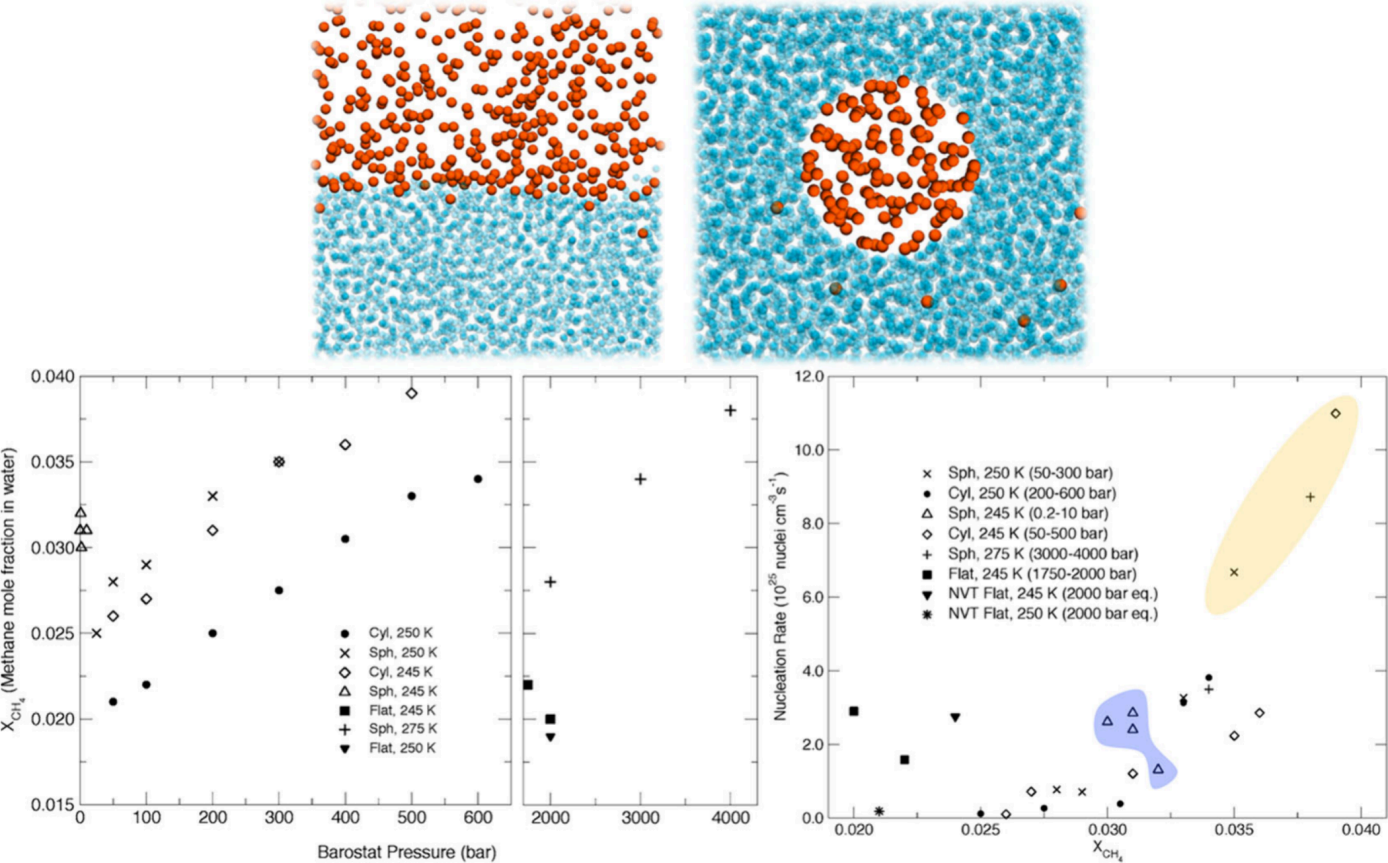
*Top*: Simulation snapshots for the systems with flat (left) and cylindrical (right) interfaces. Orange spheres symbolize methane and cyan spheres represent water oxygen atoms. *Bottom*: Methane solubility in water (left) and methane hydrate nucleation rates (right) estimated at the thermodynamic conditions and at the different interfacial curvatures investigated in Walsh et al.’s study.^75^ Reproduced from Walsh et al.^75^ Copyright 2011 American Chemical Society.

MD simulations have further indicated that this correlation still holds true even at the nanoscale.^77^ The solubility measurements for flat interfaces emphasize the substantial influence of interfacial curvature. To achieve similar methane concentrations in water at comparable temperatures (e.g., 245–250 K), systems with flat interfaces require pressures at least ten times higher than those with curved interfaces (see Figure 3, bottom left).^75^ These findings highlight the critical role of interfacial geometry in determining methane solubility.

Overall, there is a clear trend of increasing nucleation rates with higher methane solubility obtained in Walsh et al.’s simulation study, which is consistent with experimental results.^75^ Moreover, the rates computed for smaller systems with curved interfaces exhibit a consistent upward trend under varying temperature and pressure conditions (245–275 K, 0.2–4000 bar) (see Figure 3, bottom right). This pattern is consistent with both theoretical and experimental studies, which highlight guest solubility as a crucial factor in driving nucleation.^78–81^ A particularly notable case appears in simulations for the system with spherical interfacial curvature at 245 K (depicted by the shaded blue area in Figure 3, bottom right). In cases where pressures are low (∼0.2 bar), the effective pressure of gas phase is predominantly influenced by curvature of an interface. Despite this, the kinetics of hydrate nucleation in these systems follow the general trend as shown in Figure 3, bottom right, aligning with those of systems operating at pressures several orders of magnitude higher, provided that the prenucleation methane solubility is similar.

Ultimately, interfacial curvature significantly influences methane solubility and thereby nucleation rates. In simulations, achieving spontaneous hydrate nucleation within feasible time scales requires pressures at least ten times greater for systems with flat interfaces than those with curved interfaces, such as spherical or cylindrical interfaces, at the equivalent temperature.

The study of solubility of guest molecules in water encompasses a wealth of interesting physics and provides essential insights into hydrate nucleation,^72^ particularly as research in this field continues to expand. Extensive research has been dedicated to developing accurate computational approaches for solubility prediction.^82–84^ From a computational perspective, free energy calculations such as thermodynamic integration^85^ and free energy perturbation^86^ are broadly utilized.^87–89^ These approaches determine solvation free energy by calculating the free energy gradient involved in moving a guest molecule from a gas phase into a solvent. Nevertheless, a significant limitation is that they overlook guest–guest interactions in both solvent and gas phases.

Ansari et al.^90^ employed the constant chemical potential MD method to calculate carbon dioxide solubility in water under constant gas chemical potential conditions. This approach integrates a grand-canonical-like setup within a canonical system by incorporating a particle reservoir. By carefully controlling the pressure of gas phase near the liquid, they achieved a precise determination of this key parameter. Their calculated solubility values showed excellent agreement with experimental data at 423 K and 100 bar.^91^

A key advantage of this two-fluid simulation setup is its ability to provide insights into how solute molecules dissolve into the liquid via the solute–water interface, a topic of significant research interest. Defining guest–water interfaces at the molecular scale is inherently difficult because of continuous molecular motion, which causes interfacial configurations and the identity of interfacial molecules to change over time. To address this, Willard and Chandler^92^ developed a simple yet effective method for identifying interfaces. The fundamental concept starts with the instantaneous density profile at a given space-time point (**r**, *t*)­

$$\rho \left(r,t\right)=\underset{i}{\sum }\delta \left(r-r_{i}\left(t\right)\right)$$ 2

where **r**_i_(*t*) represents the location of the *i*th particle at time *t*, with the summation covering all relevant particles. Direct visualization of this density profile offers only an ambiguous representation of interfaces. To obtain a more structured and interpretable profile, coarse-graining is applied. In this approach, spatial coarse-graining is achieved by convolving with normalized Gaussian functions^92^

$$\phi \left(r;\xi \right)={\left(2\pi \xi^{2}\right)}^{-d/2}\;\exp\left(-\frac{r^{2}}{2\xi^{2}}\right)$$ 3

where *r* represents the magnitude of **r**, *d* corresponds to the dimensionality, and ξ denotes the coarse-graining length. When this process is applied to ρ­(**r**, *t*), it yields a smooth, coarse-grained density profile:^92^

$$\mathrm{\rho ̅}\left(r,t\right)=\underset{i}{\sum }\phi \left(\left|r-r_{i}\left(t\right)\right|;\xi \right)$$ 4

The instantaneous interface is characterized as the collection of spatial points where the *ρ̅*(**r**, *t*) reaches a constant value *c*. As configurations for the simulated systems evolve over time, this coarse-grained density also varies, expressed as **s** = **s**(*t*) = **s**(**r**_*i*_(*t*)). This method is adaptable to various geometries and applicable at any moment, making it particularly valuable for analyzing time-dependent phenomena and fluctuations.^92^

Using the Willard–Chandler approach,^92^ Ansari et al.^90^ analyzed the instantaneous carbon dioxide–water interface and found that its undulations result from capillary wave fluctuations (see Figure 4, top). These fluctuations significantly impact the local water structure, carbon dioxide adsorption at the interface, and carbon dioxide diffusion across it. They investigated a possible diffusion mechanism in which carbon dioxide molecules pass through transient empty patches on the instantaneous interface. Their results revealed that in the trough regions of the interface, an extended two-dimensional hydrogen bond network makes it difficult for carbon dioxide molecules to penetrate. Conversely, the larger available free area in the crest regions facilitates carbon dioxide diffusion into the bulk liquid (see Figure 4, bottom).^90^ This suggests that greater interfacial undulations create more free area in the crest regions, enhancing gas diffusion and, consequently, increasing gas solubility, which aligns qualitatively with the findings of Walsh et al.^75^

**Figure 4:**
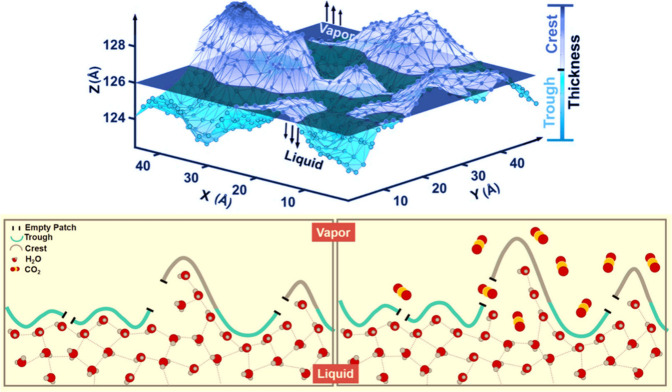
*Top*: Willard–Chandler instantaneous interface with the trough (cyan) and crest (blue) regions. *Bottom*: Schematic representation of the gas molecule diffusion across the instantaneous interface. Reproduced from Ansari et al.^90^ Copyright 2020 American Chemical Society.

*A fundamental question arises from the quantitative analysis of instantaneous guest–water interfaces: What factors could govern interfacial undulations?*

## 4 Role of Surface-Active Chemicals in Governing Gas Hydration Formation

Surface-active chemicals (SACs), also known as additives, are compounds with both hydrophobic and hydrophilic components, making them amphiphilic—allowing them to interact with both nonpolar and polar components (see Figure 5). The specific hydrophobic and hydrophilic groups present in each SAC molecule play a crucial role in determining its properties. SACs can migrate from the bulk phase to an interface, where they impact surface charge and viscosity, influence interfacial tension, alter the surface wettability, and adjust the contact angle between different phases.^93^ Their application in hydrate research dates back to the early 1990s.^94–96^ A comprehensive review of the literature highlights that SACs significantly accelerate the kinetics of hydrate formation and have been widely utilized in various lab- and industrial-scale studies.^97^ However, their use can cause foam formation during hydrate dissociation, which requires careful management.^63^ As alternatives, porous materials^98^ and nanoparticles^99^ have been introduced to hydrate systems to increase the guest–water interfacial area. While effective, their bulkiness and weight can reduce volumetric guest storage capacity and increase storage and transport costs—challenges especially relevant to hydrate-based gas storage and transport applications (see Table 1).^63^ Thus, SACs remain a preferred choice for modifying the hydrate formation and growth kinetics due to their effectiveness and scalability. However, there remains considerable uncertainty regarding which SACs are most effective for specific systems. Research suggests that the effectiveness of SACs in enhancing hydrate formation kinetics is system dependent.^100–102^

**Table 1:** Various Materials Used as Hydrate Additives to Promote Hydrate Formation and Growth

Materials used	Mechanisms governing hydrate formation	Challenges
Surface-active chemicals (e.g., surfactants, amino acids)	Enhancing the guest molecule dissolution into the aqueous phase	Cause foam formation during hydrate dissociation
Porous materials or nanoparticles	Increasing the guest–water interface area	Reduce volumetric gas storage capacity and increase storage and transportation costs

**Figure 5:**
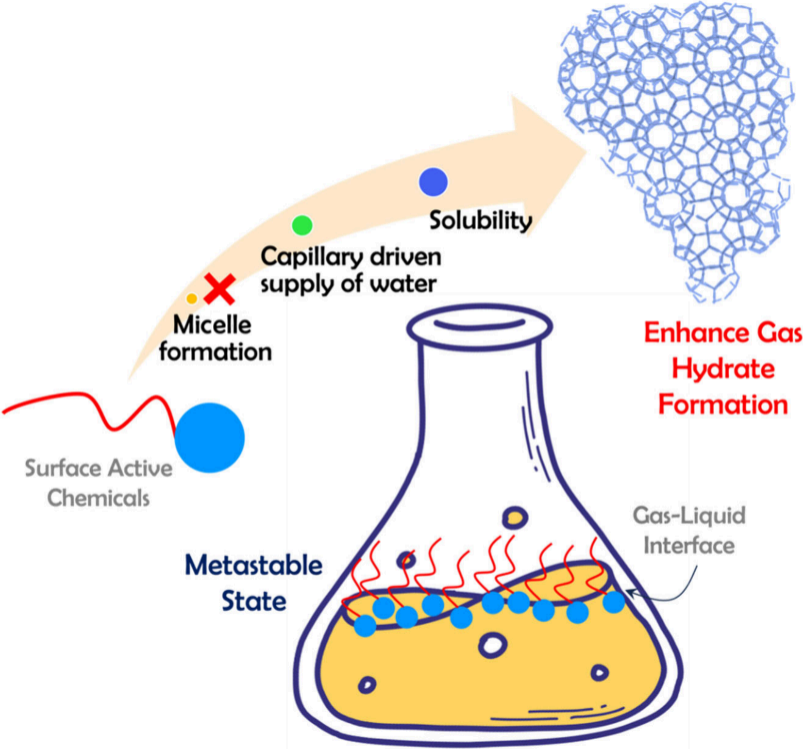
Mechanism of action of surface-active chemicals in promoting gas hydration formation.

Although substantial research has explored how SACs enhance hydrate growth, no definitive consensus on the underlying mechanisms has been reached. A detailed analysis of existing literature indicates that the increased rates of hydrate nucleation and growth might not be directly associated with the formation of micelle.^62,103,104^ Instead, other potential mechanisms include enhanced mass transport and increased gas solubility in the presence of SACs (see Figure 5 and Table 2). This raises a fundamental question: If SACs are adsorbed at the interface, how do they significantly alter gas solubility and thereby regulate gas hydrate growth at a guest–water interface? One proposed explanation is that SACs reduce adhesion energy and interfacial tension, lowering mass transport barriers and consequently enhancing gas solubility.^62^ However, previous simulation studies have reported no direct correlation between interfacial tension and hydrate growth rate, leaving this issue open for further investigation.^62,105^

**Table 2:** Multiple Approaches for Controlling Mass Transfer at Interfaces during Hydrate Formation and Growth

Mechanisms of action of SACs	Studies supporting/adopting	Comments
Micelle formation	Watanabe et al.,^103^ Savelli et al.,^104^ Phan et al.^62^	Enhanced hydrate formation may not be directly related to micelle formation
Capillary driven supply of water	Englezos et al.,^106^ Melnikov and Nesterov^107^	Enhanced hydrate formation is attributed to improved guest–water contact via porous hydrates facilitated by capillary effects
Enhanced mass transport and solubility	Zhang et al.,^108,109^ Englezos et al.,^106^ Zerpa et al.^3^	Enhanced hydrate formation is driven by increased mass transport rates at interfaces and improved guest solubility

### 4.1 SACs Induce Changes in Interfacial Undulations

Given the crucial role of instantaneous interface undulations in the diffusion of guest molecules at the guest–water interface, Phan et al.^105^ have recently investigated the surface area of the methane–water interface in the presence of disodium 1-(oleamido monoethanolamine) sulfosuccinate (DSOS), an experimentally synthesized SAC designed to enhance methane hydrate formation.^105^ To analyze this, Phan et al.^105^ applied the approach proposed by Willard and Chandler,^92^ using a water bulk density criterion (*c* = 0.5) and a coarse-graining length (ξ) of 0.24 nm.^62,110,111^ Figure 6a presents simulation snapshots showing system configurations without and with DSOS molecules at 9 MPa and 275 K. Two Willard–Chandler interfaces (bright yellow) are also presented. The ensemble-averaged interfacial surface area was determined by averaging the individual areas of Willard–Chandler interfaces for each simulated system. The change in interfacial surface area, *ΔA*, was computed by comparing it to a flat interface with the identical *X*–*Y* dimensions as^112^

ΔA=⟨A(WC)−A⟩ 5

where *A* is the flat surface area and *A*(WC) is the Willard–Chandler surface area. *ΔG*_def_ is estimated as deformation free energy responsible for the formation of instantaneous interface undulations with the following expression^113^

$$\Delta G_{def}=\gamma \times \Delta A=\gamma \times \left\langle A\left(WC\right)-A\right\rangle$$ 6

where γ is the guest–water surface tension.

**Figure 6:**
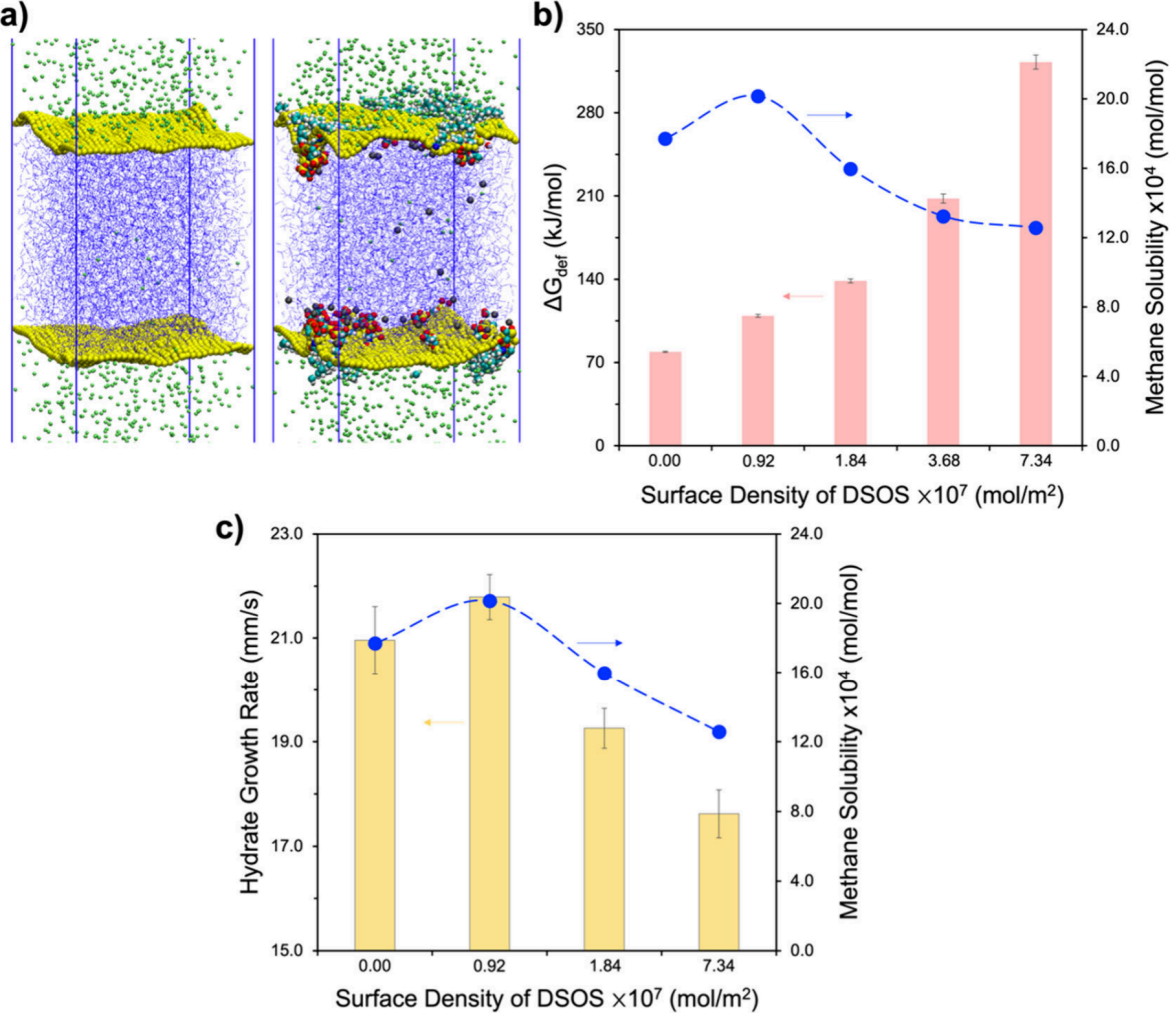
(a) Simulation snapshots for the configurations for the systems without (left) and with (right) DSOS molecules at the Willard-Chandler instantaneous interfaces (represented in bright yellow). (b) Deformation free energy *ΔG*_def_ (pink bars) responsible for the formation of instantaneous interface undulations, together with the associated methane solubility (blue circles), presented for systems without and with DSOS at different concentrations. (c) Methane hydrate growth rate at 9 MPa and 275 K (yellow bars), together with the associated methane solubility (blue circles), for systems without and with DSOS at varying concentrations. Reproduced or adapted with permission from ref 105. Copyright 2025 the Royal Society of Chemistry.

The results show *ΔG*_def_ (pink bars) alongside methane solubility (blue circles) for systems without and with DSOS. The *ΔG*_def_ value peaks at 7.34 × 10^–7^ mol/m^2^ of DSOS. This suggests that the addition of DSOS leads to higher *ΔG*_def_ (or larger *A*(WC)), aligning with previous observations of increased oil–water interfacial roughness in the presence of sodium dodecyl sulfate^114^ and tri-*n*-butyl phosphate^115^ surfactants. Although a higher *ΔG*_def_ might be expected to facilitate methane transport, thereby increasing methane solubility, these findings indicate no clear correlation between *ΔG*_def_ and methane solubility which is directly linked to the rates of hydrate growth for the systems analyzed (see Figure 6b). It is important to note that the simulated methane hydrate growth rates and corresponding methane solubility at varying DSOS concentrations (Figure 6c) qualitatively align with the experimental results,^105^ which show highest methane uptake at 500 ppm DSOS, with no notable increase when the DSOS concentration is further increased.

These insights suggest that additional factors might counteract the effect of the increased interfacial undulations induced by SACs in enhancing gas solubility (see Table 3). *It is essential to highlight that the addition of SACs may introduce steric hindrance, thereby raising barriers to diffusion of methane into the aqueous phase.*

**Table 3:** Role of SACs in Governing the Kinetics of Gas Hydration Formation and Growth

Actions of SACs	Comments	Studies supporting/adopting
Changes in interfacial undulations	Higher concentrations of SACs lead to increased interfacial roughness (greater *ΔG*_def_), which enhances solubility	Bui et al.,^114^ Clark et al.,^115^ Phan et al.^62^
Changes in mass transfer thermodynamic at guest–water interface	Higher concentrations of SACs raise the free energy barrier for guest molecule diffusion, thereby decreasing solubility	Sicard et al.,^116^ Farhadian et al.,^105^ Phan et al.^62^

### 4.2 SACs Cause Thermodynamic Changes in Guest Molecules at Interfaces

Sicard et al.^116^ examined this phenomenon by quantifying the effectiveness of a thin film of anti-agglomerants in restricting methane transport from the hydrocarbon phase to the growing hydrate (see Figure 7A). The studied anti-agglomerant was a SAC featuring hydrophobic tails and an intricate hydrophilic head containing tertiary ammonium cation and amide groups.^8,117^ Sicard et al.^116^ conducted well-tempered metadynamics simulations^118,119^ to identify the minimal free energy pathway. Figure 7B presents the free energy pathways accompanied by snapshots illustrating various scenarios. The identified free energy pathways take place in interfacial regions with varying anti-agglomerant surface densities. The minimal (black), intermediate (red), and maximal free energy pathways (blue) correspond to methane diffusion through interfacial regions with increasing anti-agglomerant surface densities. Due to the rigidity of anti-agglomerant molecules the simulations used to obtain maximal free energy pathways, methane diffusion through a densely packed anti-agglomerant region faces a significant free energy barrier.^116^

**Figure 7:**
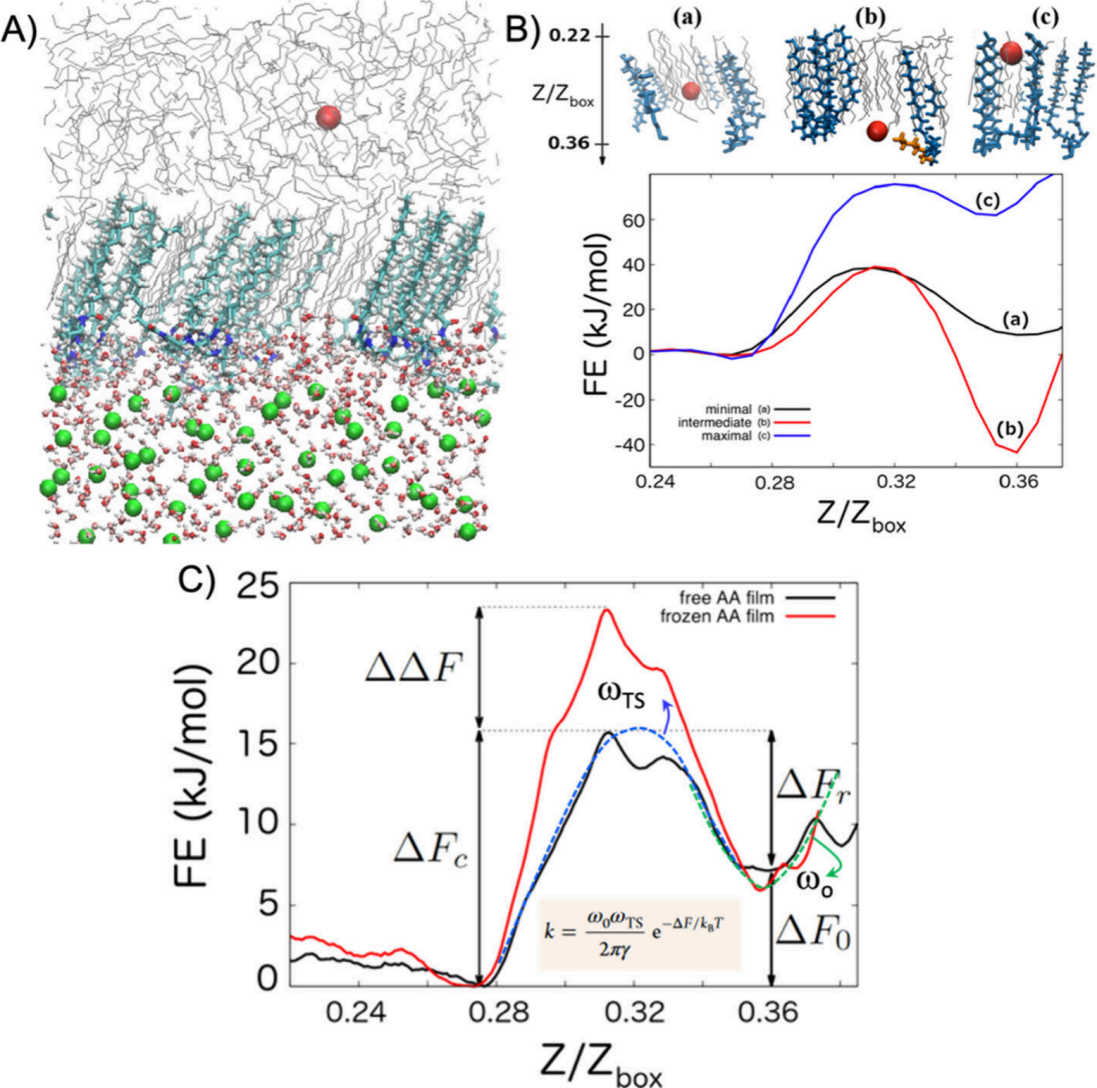
(A) Simulation snapshots for the configuration for the system at anti-agglomerant surface density of 0.67 molecule/nm^2^. The free methane molecule, analyzed for free energy pathways, is represented as a red sphere. (B) Top: Schematic representation of the minimal (a), intermediate (b), and maximal (c) scenarios. Gray lines depict n-dodecane, while blue line indicate anti-agglomerant molecules. Bottom: Free energy pathways obtained with the well-tempered metadynamics simulation techniques. (C) Free energy pathways for the single methane molecule moving across the interface when the anti-agglomerant molecules are either free (black) or frozen in position (red). Reproduced from Sicard et al.^116^ Copyright 2018 American Chemical Society.

Employing the umbrella sampling^120^–adiabatic biased MD^121–123^ framework, Sicard et al.^116^ then compared the free energy pathways obtained under two different scenarios: (1) fixed anti-agglomerant layer—the anti-agglomerant layer remains stationary; (2) flexible anti-agglomerant layer—the anti-agglomerant molecules can move. In both scenarios, the anti-agglomerant molecules induced rigidity and alignment of hydrocarbon molecules (e.g., dodecane) at the hydrocarbon–growing hydrate interface, consistent with findings by Bui et al.^8^ Restricting the movement of the anti-agglomerant molecules did not alter the free energy Δ*F*_0_ ≈ 8.5 kJ/mol or the positions of the local and global minima (*Z*/*Z*_box_ = 0.36 and *Z*/*Z*_box_ = 0.27, respectively), as these regions above and below the interfacial layer are devoid of anti-agglomerant molecules.^116^ However, freezing the anti-agglomerant molecules increased the free energy barriers, with ΔΔ*F* ≡ Δ*F*_c,frozen_ – Δ*F*_c,free_ ≈ 7.5 kJ/mol at the transition point^116^ (*Z*/*Z*_box_ ≈ 0.31, see Figure 7C).

These simulation results suggest that a higher surface density of SACs at interfaces generally raises the free energy barrier, ultimately enhancing the effectiveness of the interfacial SAC layer in inhibiting the diffusion of guest molecules (see Table 3).

## 5 Significance of Thermodynamic Analysis at Interfaces in Hydrate Research

Inspired by Prausnitz’s commentary “*The fascination of any growing science lies in the work of the pioneers at the very borderland of the unknown. But to reach that frontier one must pass over well-travelled roads. One of the safest and surest is the broad highway of thermodynamics*”,^124^ Phan et al.^62^ draw upon the thermodynamic pathway to provide fundamental understanding of how SACs influence hydrate formation and growth at interfaces, aiming to pioneer this evolving field.

Specifically, Phan et al.^62^ performed thermodynamic calculations to explore the interactions among methane, SACs (sodium dodecyl sulfate (SDS) and polyvinylcaprolactam oligomer (CAP)), water, and hydrocabon solvents in relation to methane solubility. The left panel of Figure 8 presents potential of mean force profiles derived from umbrella sampling calculations.^120^ These profiles represent the interactions experienced by a single methane molecule moving across the hydrocarbon–water interface toward the aqueous phase. The results are plotted as a function of the distance *l* between the interface and the methane molecule. In the absence of SACs or at low SAC surface densities (green), the potential of mean force profiles reveal an effective attraction between methane and interfacial water molecules. This is followed by a repulsive barrier, likely caused by disruptions in the water hydrogen-bonding network near methane. Nevertheless, at high SAC surface densities, the potential of mean force profiles display a repulsive barrier at intermediate *l*, followed by a minimum, and a significantly greater repulsive barrier near the interface.^62^ These findings qualitatively align with previous results,^116,125^ which reported higher free energy barriers for methane diffusion as SAC surface densities at the interface increased.

**Figure 8:**
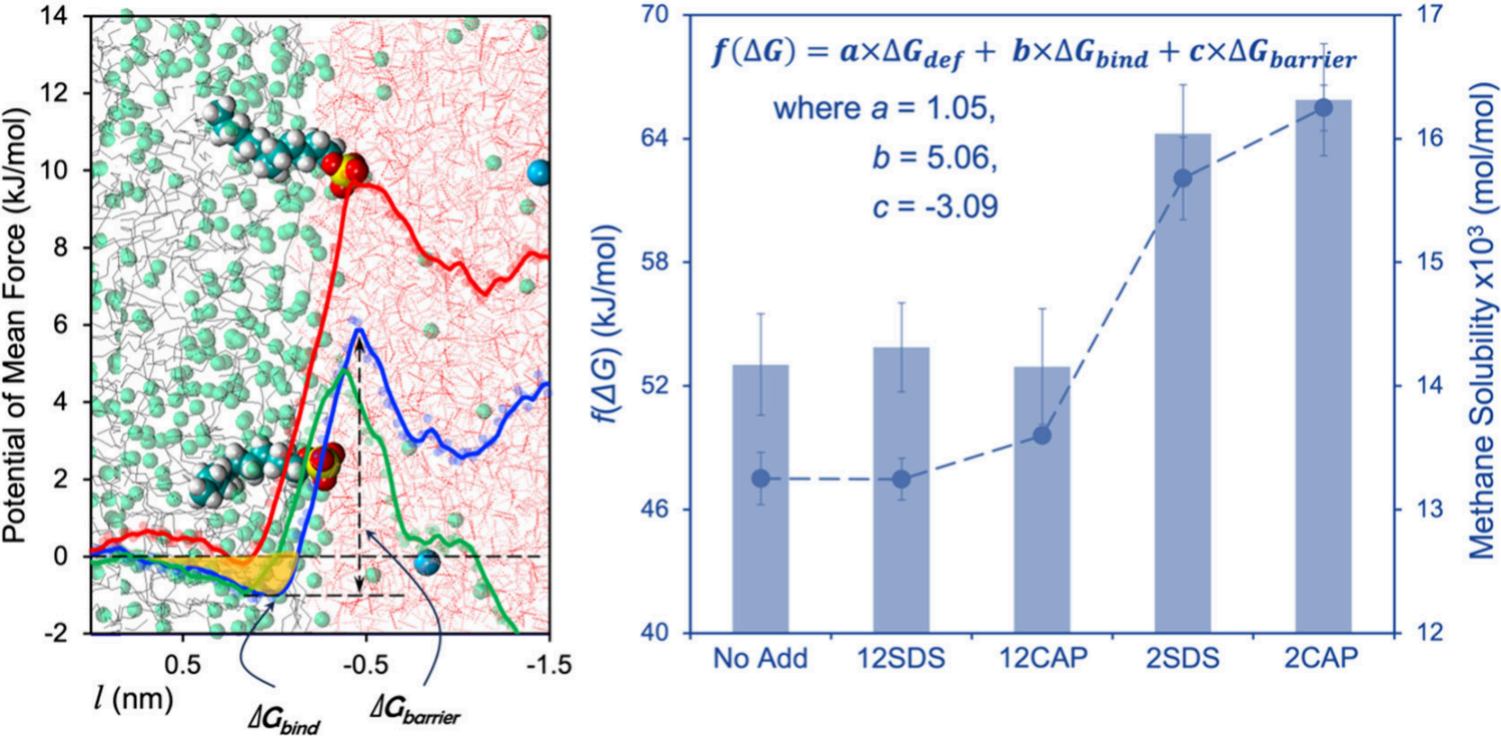
*Left*: Potential of mean force profiles for a single methane molecule migrating from the hydrocarbon phase to the aqueous phase. The results were shown for simulated systems without (blue) and with SACs at low (green) and high (red) surface densities. *Right*: Fitting data for the deformation free energy (*ΔG*_def_), the adsorption barrier (*ΔG*_barrier_), the effective binding free energy (*ΔG*_bind_) to a multiple linear regression model associated with corresponding methane solubility (circles) achieved for systems without and with SACs (SDS and CAP) at varying surface densities at the interface. Reproduced from Phan et al.^62^ Available under a CC-BY 4.0 license. Copyright 2023 Elsevier B.V.

Phan et al.^62^ further applied the multilinear regression model to fit the deformation free energy (*ΔG*_def_), the adsorption barrier (*ΔG*_barrier_), the effective binding free energy (*ΔG*_bind_)—obtained by integrating the potential of mean force profiles within the attractive region— for all systems studied:

$$f\left(\Delta G\right)=a\times {\Delta G}_{def}+b\times {\Delta G}_{bind}+c\times {\Delta G}_{barrier}$$ 7

A strong correlation between *f*(*ΔG*) and methane solubility was observed, with coefficients *a* = 1.05, *b* = 5.06, and *c* = −3.09 (see Figure 8, right panel). These findings suggest that potential of mean force profiles for methane diffusion (*ΔG*_barrier_ and *ΔG*_bind_) along with the deformation free energy *ΔG*_def_ offer valuable insights into gas transport through the hydrocarbon–water interface. Consequently, these parameters can serve as indirect predictors for quantifying and potentially forecasting the hydrate formation and growth rates.^62^ This demonstrates that a detailed mass transfer thermodynamic analysis through a guest–water interface could unveil molecular-level mechanisms through which SACs function as effective promoters of hydrate formation and growth.

## 6 Challenges in Elucidating the Hydrate Formation and Growth Mechanism at Interfaces

Although considerable efforts have been made, hydrate-based technologies for gas storage, separation, and transport remain unrealized, primarily due to the slow kinetics of hydrate formation and growth. These kinetics are hindered by thermodynamic and kinetic barriers, including stochastic nucleation and constraints in mass and heat transfer.^63^ One major challenge lies in the intrinsically unpredictable nature of nucleation, which prevents hydrate formation within a reasonable time frame.^126^ Recent simulations indicate that the nucleation of hydrate under nonsupercritical conditions occurs on time scales of microseconds,^42,60,127,128^ making it difficult to capture statistically. Key challenges in the simulations of hydrate nucleation include the use of excessively high supersaturation levels, accurate phase diagram predictions, and the selection of suitable observables.^126^ Although the mechanisms of hydrate growth are somewhat clearer than nucleation, understanding mass transfer at guest–water interfaces is critical to both processes. Larger systems are needed to model phenomena at interfaces, avoiding finite-size effects and the need for longer simulations.^126^ Additionally, the misuse of artificial thermostats in MD simulations, particularly at interfaces, can artificially smooth temperature gradients and distort heat conduction. English and MacElroy,^129^ along with Kusalik and Vatamanu,^130,131^ have tackled this issue by applying local thermostats to different regions within the simulation system.

Several approaches have been suggested to improve mass transfer, including the use of SACs as hydrate promoters to enhance guest molecule dissolution into the bulk water phase.^62,63,97,105^ However, accurately modeling water, guest, and SAC interactions remains a challenge, especially for hydrate systems. Few simulations have used potential models specifically parametrized for water–guest–SAC systems. Recent interest in environmentally friendly SACs has sparked efforts to design molecular architectures for accurate MD simulations. This involves developing accurate force fields, utilizing advanced ab initio MD simulations, and fitting potentials to forces rather than potential energy surfaces.^126^ Analyzing free-energy profiles can help evaluate SACs as hydrate additives, and combining molecular simulations with experimental mass transfer thermodynamics could guide the design of SACs for hydrate-based technologies.^62,105^

## 7 Future Directions

### 7.1 Connections between Theoretical and Experimental Efforts in Mass Transfer Thermodynamics

The potential of mean force profiles, also known as free energy profiles, that govern the transport of guest molecules across a guest–water interface have been extensively studied through computational modellings.^133–137^ Experimental investigations for the free energy profile at this interface, however, requires distinguishing between guest–interface and guest–aqueous thermodynamics. While the thermodynamic properties of gas–bulk liquid are commonly measured, directly assessing the surface thermodynamics of interactions between a liquid interface and gas-phase species under in situ conditions remains a significant technological challenge.^138^ Currently, direct molecular-level assessments of gas–liquid interfaces using analytical surface characterization methods^139,140^ to determine equilibrium surface free energy, entropy, and enthalpy are limited. This is largely because analyzing a gas–liquid interface with chemical and atomic precision while simultaneously adjusting gas-phase pressure and temperature under equilibrium conditions is highly challenging. To address this, Broderick et al.^132^ employed ambient pressure X-ray photoelectron spectroscopy (AP-XPS)^141–143^ and gravimetric analysis to estimate the free energy, entropy, and enthalpy of gas-phase water at the gas–ionic liquid interface. Their findings indicate that the entropy and enthalpy of water adsorption differ remarkably between the bulk and the gas–ionic liquid interface, depending on the reaction coordinate. Free energy calculations further indicate that water at the gas–ionic liquid interface is energetically more favorable than in the bulk, aligning with the increased water concentration observed in the interfacial area.^132^

These in situ measurements using AP-XPS and gravimetric analysis could be instrumental in experimentally investigating the diffusion of guest molecules from the hydrocarbon phase to the aqueous phase. Furthermore, they can help validate the thermodynamic variations and structural dynamics across the guest–water interface predicted by MD simulations and free energy calculations (see Figure 9). This probably offers a more profound understanding of the molecular-level mechanisms underlying hydrate formation and growth at guest–water interfaces, ultimately contributing to advancements in hydrate-based applications and strategies for enhancing hydrate formation.

**Figure 9:**
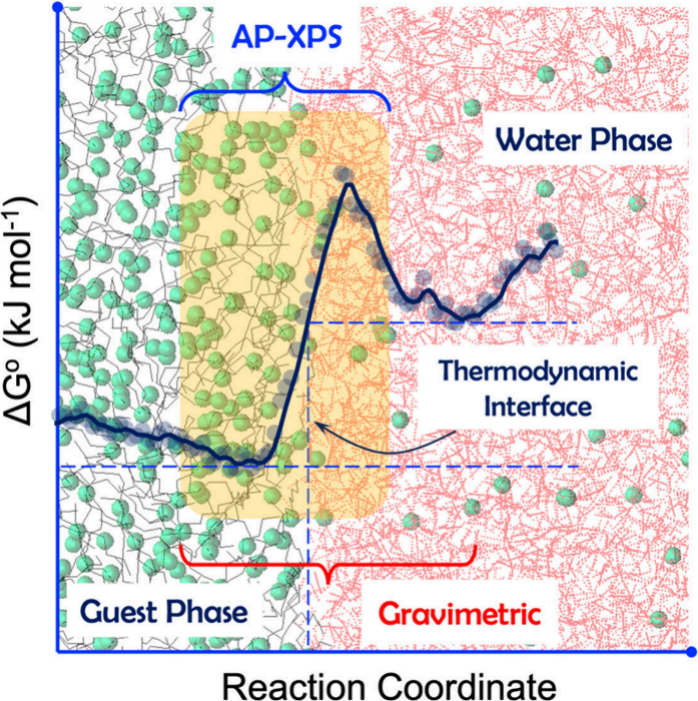
Variations in surface thermodynamics across a guest–water interface. Reproduced from Broderick et al.^132^ Copyright 2019 American Chemical Society.

### 7.2 Developing Next Generation Hydrate Inhibitors and Promoters

For over two decades, low-dosage hydrate inhibitors, such as anti-agglomerants and kinetic hydrate inhibitors, have been widely examined.^144^ While anti-agglomerants primarily affect the hydrate growth phase, kinetic hydrate inhibitors, primarily polymeric compounds like polyvinyl caprolactam and polyvinylpyrrolidone, influence the hydrate formation and growth stages. The development and selection of both kinetic hydrate inhibitors and anti-agglomerants for industrial hydrate prevention should adhere to three key criteria: enhanced effectiveness, environmental compatibility, and cost efficiency. Commercially viable kinetic hydrate inhibitors and anti-agglomerants should possess these properties while effectively slowing the hydrate growth kinetics by adsorbing onto the interface surface.

These principles can also be applied to the evaluation and selection of hydrate promoters. Emerging engineering applications utilizing clathrate hydrates—such as seawater desalination, hydrogen storage, and natural gas storage—demand efficient SAC promoters to accelerate the hydrate formation and growth. For example, hydrogen storage, is a critical challenge for next-generation clean energy technologies. The commercial viability of storing hydrogen in hydrate structures is currently hindered by the requirement of extremely high pressures (>2300 MPa) at ambient temperatures.^145,146^ SACs have the potential to enhance mass transfer by improving water–gas mixing, thereby accelerating hydrate crystal growth and facilitating more practical hydrogen storage solutions.

Molecular-level surface spectroscopy measurements and molecular simulations^62,73,116,132^ indicate that the interfacial region is pivotal in regulating the mass transfer of guest molecules interacting with SACs, hydrocarbons, and aqueous phases. The adsorption and movement of these molecules across the interface are governed by surface thermodynamics.^62,132^ Since the kinetics of hydrate formation/dissociation depend on the transport process of guest molecules into the bulk aqueous phase via the guest–water interface, computational studies have suggested a correlation between hydrate kinetics and thermodynamic variations in mass transfer across the interface.^62^

Although experimental methods exist for studying mass transfer thermodynamics,^132^ their application to high-throughput screening of potential SAC candidates for hydrate inhibition or promotion is impractical due to the significant time and cost required to explore the vast chemical space of small molecules. MD simulations provide an alternative, in silico approach to examine structural dynamics driving the thermodynamic variations at interfaces. Enhanced sampling techniques such as umbrella sampling^120^ and metadynamics^118,119^ have been widely employed to obtain free energy profiles for gas-phase molecules entering bulk liquids across gas–liquid interfaces. However, the computational demands of MD restrict its feasibility to studying only hundreds or thousands of molecules within a realistic time frame, even with coarse-grained modeling techniques.^147^ Current advancements in machine learning offer a promising solution by enabling high-throughput calculation of pharmaceutical and physicochemical properties.^148^ This process typically involves extracting molecular fingerprints and descriptors using cheminformatics tools, developing predictive models with statistical learning techniques, and validating these models on diverse data sets. While machine learning significantly reduces computational costs compared to MD simulations, it has limitations such as sparse training data, low interpretability, moderate accuracy, and limited transferability.

An integrated computational framework that combines MD simulations with machine learning can mitigate these limitations by leveraging the strengths of both approaches. Physical insights derived from MD simulations can enhance the interpretability and precision of machine learning predictions while enabling the high-throughput screening of thousands of molecules. Several studides proposed that modifying the structure and composition of the groups connected to the amino and carboxylic acid functional groups of amino acids can significantly influence their performance as hydrate additives—either enhancing or suppressing their activity.^149^ For instance, l-tryptophan has shown the highest effectiveness as a promoter for the formation of methane hydrate, likely due to its aromatic side chain and hydrophobic character.^39^ Furthermore, combining different types of SACs has shown potential in enhancing hydrate formation kinetics more effectively than using a single SAC alone (e.g., a mixture of 1,3-dioxane and l-leucine improved hydrogen hydrate formation).^38^ However, such combinations may also exhibit antagonistic effects depending on the specific molecular structures involved.^35^ Inspired by these findings, MD fingerprints^150^ could be proposed to characterize the molecular features of SACs, using amino acids as initial models. To encode thermodynamic properties such as free energy, entropy, and enthalpy, descriptors—including the average, median, and standard deviation of features like hydrophobic chain length, the nature of hydrophobic/hydrophilic groups, potential energy components, interfacial surface area, SAC concentrations, and SAC combinations—can be extracted from short MD simulations at the guest–water interface. Combined with simple 2D molecular descriptors, these fingerprints have been used to train machine learning models to predict key properties such as guest solubility, deformation free energy, adsorption barrier, and effective binding free energy. This approach has demonstrated improved predictive accuracy while remaining less computationally intensive than traditional MD-based methods, thus drawing significant interest.^151^ In addition, MD-generated data can supplement training data sets when experimental data is unavailable. This hybrid approach offers a powerful means for massively parallel virtual screening of molecules using mass transfer thermodynamics, highlights the potential of language processing techniques in gas hydrate research, and demonstrates an innovative integration of physics with statistical learning for commercial applications.

## 8 Conclusions

Knowledge of gas hydrate structures and their physicochemical properties is rapidly advancing. Gaining thorough insights into the hydrate crystal formation and growth is essential, as it shapes both fundamental hydrate-driven science and engineering technologies. This mini-review seeks to offer an in-depth overview of clathrate hydrates, focusing on the fundamental molecular-level mechanisms of hydrate formation and growth at guest–water interfaces. Hydrate formation and growth are inherently governed by free energy, affected by temperatures, and occur as statistically random processes, with a higher probability at interfaces where favorable driving forces are present. Recognizing the current state of knowledge, identifying gaps, and exploring areas for improvement are vital for advancing research in this field. This awareness not only deepens our core knowledge but also lays the foundation for future advancements in hydrate science and engineering.
